# Assessment of Water Quality Parameters in Lake Hayq, Northeastern Ethiopia

**DOI:** 10.1155/2024/7439024

**Published:** 2024-09-04

**Authors:** Assefa Tecklie, Yohannes Nigussie, Adem Bilale

**Affiliations:** ^1^ Wollo University Department of Biology, Dessie, Ethiopia; ^2^ Ministry of Water and Energy Ecohydrology and Water Quality Desk, Addis Ababa, Ethiopia

## Abstract

Lake Hayq is one of the highland lakes of Ethiopia that furnishes very important ecosystem services, fishing, tourism, transportation, drinking water, livestock watering, and irrigation. However, the lake ecosystem is being degraded by pollution, siltation, and excessive growth of macrophytes, buffer zone degradation, overfishing, and climate variability. Therefore, this study was conducted to assess the physicochemical, heavy metals, and biological water quality parameters of Lake Hayq. Physiochemical (pH, water temperature, conductivity, TDS, total alkalinity, dissolved oxygen, Ca^2+^, Mg^2+^, Na^+^, K^+^, NH4^+^, NH_3_, NO_2_^−^, NO_3_^−^, CO_3_^−^, HCO_3_^−^, SO_4_^2−^, PO_4_^3−^, SiO_2_, and total phosphorus), heavy metals (Pb, Fe, Zn, Cr, Cu^2+^, Mn^2+^, and Ni), and biological (BOD_5_ and total coliforms) water quality parameters were analyzed both in situ and ex situ. The physicochemical parameters were measured using portable water quality measuring multimeters, the heavy metal analysis was done using the Atomic Absorption Spectrometer, the BOD_5_ was measured using a BOD_5_ meter, and the total coliform analysis was done using the spread plate technique. The collected data were analyzed using multivariate, two-way ANOVA to see the mean difference among sampling sites and seasons through the application of SPSS 16. Most of the water quality parameters of Lake Hayq have met the WHO standards for recreation, aquatic life, and drinking water quality. However, some parameters, such as Pb, BOD_5_, and total coliforms, were above WHO water quality permissible limits. Therefore, ecohydrological (nature-based) waste treatment methods such as macrophyte restoration in buffer zones and ecofriendly farming activities should be practiced to minimize the contamination of the lake.

## 1. Introduction

Water is one of the most important ecosystem services supporting human beings regularly given support to human being in different ways. For instance, it is used for food production, fishery, transportation, recreation, cultural, religious activities and other human activities.

However, in the last few decades, the intensive utilization of water resources for various activities contributes to the pressure on their availability [1, 2] and causing negative effect on the water quality [3, 4].

Lakes are among the freshwater bodies which have been impaired with nutrients from natural and mainly man-made sources. The recent expansion of urbanization, industrialization, agricultural activities, and climate change are the major causes of nutrient enrichment, eutrophication globally, causing water degradation, loss of its uses, and water-associated economic and health effects [5, 6].

The evaluation of water quality of lakes is very essential due to its significant role in terms of ecosystem services. However, anthropogenic pressures such as rapid urbanization, surface runoff of waste water from agricultural activities, and land use land cover change are degrading water quality that needs better attention to the causes of pollution and their controlling mechanisms [7].

The quality of water determined commonly with their physical, chemical, and biological parameters [8, 9]. The deterioration of water quality has been an upsetting issue in poor communities where the treatment of waste before being discharged into the water bodies is of low priority [10, 11].

Contamination of freshwater with heavy metals due to natural processes such as atmospheric accumulation and geological weathering, as well as anthropogenic activities (domestic, urban waste, industrial effluents, and runoff from agricultural activities), are great concerns in both developing and developed nations [12]. Lakes are among the freshwater bodies usually vulnerable the contamination of these metals as they are relatively stagnant in nature compared to rivers and streams, which have more self-purification potential [13, 14].

Heavy metals such as chromium (Cr), copper (Cu), manganese (Mn), nickel (Ni), lead (Pb), and zinc (Zn) are among the major contaminants in aquatic systems that have high toxicity to human health if their concentrations exceed the standard [15, 16].

In the aquatic ecosystem, chromium (Cr), copper (Cu), manganese (Mn), nickel (Ni), lead (Pb), and zinc (Zn) are the major trace metals commonly contaminating water. Of these trace elements, Cr, Pb, and Zn have both carcinogenic and noncarcinogenic risks to aquatic life and public health concern [17–19]. Therefore, the assessment of these metals and their associated potential risk to lake water quality is very important from a human and ecological health perspective [16, 20].

Globally, coliform bacteria are used as indicators of environmental and fecal contamination and, hence, the possible presence of pathogenic organisms [21, 22]. Inland water bodies, stream, and lake monitoring involve assessing the microbial quality of water and the risk of possible transmission of waterborne infectious diseases.

Similar to other lakes found in other countries globally, lakes of Ethiopia have ecological and economic services; however, they are being affected by pollution or eutrophication due to agriculture, urbanization, and industries, which have increased over the past few decades in Ethiopia [23]. According to Fetahi [23], eutrophication has become a threat for many lakes located in major towns and cities and in areas where agricultural activities are more in Ethiopia. Other scholar has also reported similar problems in Ethiopian lakes. For instance, eutrophication has been reported from Lake Zeway [24, 25] Lake Tana [26], and Lake Hawasa [27].

Lake Hayq is one of these kinds of lakes highly affected by pollution [28], siltation [29, 30], land use and land cover change [29], and natural factors such as rainfall variability and temperature increment [31].

Many of the scientific studies reported so far in Lake Hayq were mainly concentrated on plankton and fish ecology [32, 33]. There is very limited information on heavy metals, organic pollution load (BOD_5_), and coliforms. Therefore, this study aimed to evaluate the water quality of Lake Hayq using some biophysicochemical water quality parameters.

## 2. Materials and Methods

### 2.1. Study Area

**Figure 1:**
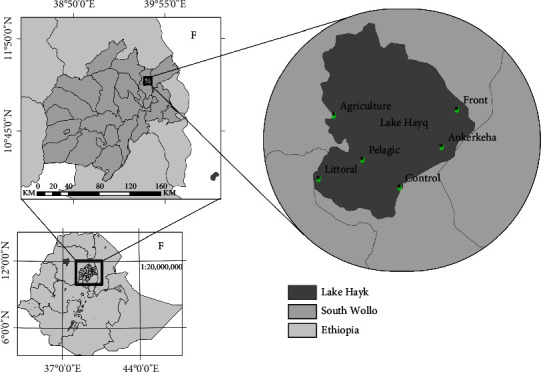
Map of Lake Hayq and the sampling sites (littoral, agriculture, front, Ankerkeha, control, and pelagic).

The study was conducted at Lake Hayq. Lake Hayq is located in the highlands of Ethiopia. It lies between 11°3′N to 11° 18′N latitude and 39° 41′E to 39° 68′E longitude, with an average elevation of 1911 meters above sea level (Figure 1). The lake has a closed drainage system, and the total watershed area is about 77 km^2^, of which 22.8 km^2^ is occupied by Lake Hayq. The average and maximum depths of the lake are 37 and 81 m, respectively [34, 35].

### 2.2. Sampling Methods

Six sampling sites (S1, S2, S3, S4, S5, and S6) were selected based on the impact of anthropogenic and livestock activities and the horizontal zonation of the lake. Site1 (SS1, littoral) has more human activities related to recreational activities in and around the lodges constructed on the shore of Lake Hayq. The second site (SS2, Agriculture) is highly exposed to agricultural activities. The third site (SS3, Front) is a highly degraded part of the lake characterized by high steepness and poor vegetation coverage. The fourth site (SS4, mouth of the Ankerkeha River, carrying huge silt every year). The fifth site (SS5, Control) is a relatively vegetated area and less impacted. The sixth site (SS6, Pelagic) is open water, which has relatively less impact from human and livestock activities (Table 1). The sampling sites were fixed with GPS, and a map was generated (Figure 1). Seasonal sampling between July 2018 and May 2019 was conducted during the four seasons: rainy (July, August, and September), postrainy (October, November, and December), dry (January, February, and March), and prerainy (April, May, and June). The sampling was done at all sampling stations at different depths. Temperature, conductivity, pH, and dissolved oxygen were measured in situ using a portable multiprobe (Wagtech Portable Water Quality Kit). Water transparency was estimated using a standard Secchi disk.

Water for chlorophyll-a (Chl-a) and nutrient analysis were sampled at the surface and different depths and were transferred and stored under an icebox until analyses were made in the Biology Department, Addis Ababa University, Ethiopia. Total alkalinity, soluble reactive phosphorus (SRP), ammonia (NH_3_), ammonium (NH_4_^+^-N), (NO_3_-N), nitrite (NO_2_-N), reactive silica (SiO_2_), and total phosphorus (TP) were analyzed following the standard procedure [36].

#### 2.2.1. Major Cations, Anions, and Heavy Metal Analysis

Water samples for major ion, heavy metal, and major anion analysis were collected from the surface water of six sampling sites and immediately transported to Addis Ababa University, Department of Earth Science Isotope Hydrology Laboratory, using clean sampling bottles.

Major cations Calcium (Ca^2+^), Magnesium (Mg^2+^), Sodium (Na^+^), Potassium (K^+^), and heavy metals (Lead (Pb), Copper (Cu), Zinc (Zn), Chromium (Cr), Manganese (Mn), Nickel (Ni), and total Iron (Fe)) were analyzed with the Atomic Absorption Spectrometer, 5oB method. Major anions Bicarbonate (HCO_3_^−^), Sulphate (SO_4_^2−^), and Chloride (Cl^−^) were analyzed using the UV Spectrophotometer Lambda EZ 201 (Double Beam).

#### 2.2.2. Biological Data

*(1) Biochemical Oxygen Demand for Five Days (*BOD*_5_) and Total Coliform Analysis*. Water samples for both BOD_5_ and total coliforms were purposely collected from a littoral site (near lodges), which is suspected of organic pollution and human and livestock contamination. The duplicate water samples were collected with properly cleaned sampling bottles during the prerainy, rainy, postrainy, and dry seasons between July 2018 and May 2019. The samples were transported immediately to the Biology Laboratory, Wollo University, Ethiopia, using an icebox.

*(2) *BOD*_5_ (Biochemical Oxygen Demand for Five Days)*. In the laboratory, the water samples were transferred to BOD_5_ bottles and incubated at 20°C for 5 days. At the end of five days, the dissolved oxygen (DO) content of the incubated bottles was read to calculate the BOD5 value using the following formula:

(1) $$\begin{array}{c} {\text{BOD}}_{5}\left(\text{mg}/L\right)=\left[\left(\text{Initial DO}-\text{Final DO}\right)\times 300\right]/\text{mL sample}. \end{array}$$

*(3) Total Coliform Analysis*. Selective media (Violet Red agar), which is specifically used for the isolation and identification of total coliforms, was prepared, and serial dilution (10^−1^–10^−6^) was done using the collected water samples. After serial dilution, 0.1 mL of a well-mixed diluted sample was inoculated aseptically on the agar surface and was distributed evenly over the surface of the medium (Violet Red Bile agar) using a sterile spreader device. The plates that contain the inoculated sample were incubated at 35°C for 48 hrs. After a 48-hr incubation period, colonies grown on Violet Red Bile agar plates were counted via a digital colony counter; only colonies that had a red to pink color were considered.

### 2.3. Data Analysis

The data on water quality parameters that were gathered were conducted using Microsoft Excel version 2010. The water quality data difference among sampling sites and seasons was analyzed using the SPSS (version 26).

## 3. Results and Discussion

### 3.1. Physicochemical Water Quality Parameters

Multivariate, two-way ANOVA analysis showed that there was a significant difference in DO, oxygen saturation, water Temperature, pH, total alkalinity, conductivity, and TDS among sites and seasons (ANOVA, *P* < 0.05). However, there was no significant difference in Secchi disk depth and turbidity (ANOVA, *P* > 0.05) in Lake Hayq. Lake Hayq is a slightly alkaline system with a mean total alkalinity of 8.4 meqL^−1^ and a pH range of 7.5–9.8. Electrical conductivity also fluctuated little, with a mean of 920 *µ*S·cm^−1^. The minimum dissolved oxygen (DO) recorded was 6.29 mgL^−1^ (91.5% saturation) during the prerainy season at 20-meter depth in the pelagic part of the lake. The mean water temperature, Secchi disk depth, turbidity, and TDS were 23.5°C, 3.12 m, 4 NTU, and 676.4 mg/l, respectively.

The DO in the present study was above 6 mg/l, higher than other similar highland lakes in Ethiopia, such as Lake Tana (DO < 5 mg/L; [37]). But it was close to the nearby highland lake, Lake Ardibo (6.51–8.53 mg/l; [30]). These differences in DO might be due to less pollution and turbidity compared to Lake Tana, which has had more siltation and water hyacinth infestations recently.

The DO is the most important parameter for aquatic life. It is the soluble form of oxygen found in lakes and reservoirs. Low levels of DO have both direct and indirect effects on the use of lakes and reservoirs. The amount of DO in the water directly affects aquatic life. Many aquatic insects, fish, and other organisms become stressed and may even die when DO levels drop below a particular threshold level (e.g., below 5 mg/l) [38–40].

The pH of Lake Hayq ranged from 7.5 to 9.98, similar to Lake Tana with a pH range of 6.98–9.97 [41]; 7.9; [37, 42]; and Lake Ardibo (9.20–9.77; [30]). However, this value was higher than Tadesse Fetahi et al.'s [33] report for the same lake and Lake Peru in India, with a pH value of 6.6–8.05 [43]. According to the Ethiopian EPA [44], the pH value should be ranged between 6.5 and 9 for safe aquatic life. Based on this guideline, the pH of Lake Hayq was slightly higher than the recommended value.

### 3.2. Inorganic Nutrients

There was a significant difference in inorganic nutrients (NO_2_^−^, NO_3_^−^, NH_3_, SiO_2_, SRP, and TP) among sites and seasons (*P* < 0.05) (Tables 2 and 3). Most of the nutrients had the highest values at Site 1 (Littoral) and Site SS4 (Ankerkeha), which might be associated with more human activities (heavy detergent loads from bathing, cloth and car washing, and waste released from lodges) and siltation loads brought through the Ankerkeha River.

The concentration of NH_3_, TP, and SRP was highest during the prerainy season which might be associated with rainfall availability, causing flooding of the Ankerkeha River that brings siltation during rainfall. The lake area has bimodal rainfall availability; rainfall was available in the prerainy season, during our sampling. However, the concentrations of NO_3_^−^ and SiO_2_ were highest during dry and postrainy seasons, respectively (Table 3).

In the present study, the concentration of limiting nutrients (nitrogen and phosphorus forms) was below the WHO standards for aquatic life and drinking water permissible limits (Table 4). The concentrations of TP and SRP were higher at the Ankerkeha River, which might be associated with siltation. These values were higher than those of Lake Wonchi, Dendi, and Ziquala [46]. But they were lower than Lake Tana [26, 41], Lake Zeway [24, 25], Lake Hawasa [27], and Tadesse et al. [33] for the same lake (Lake Hayq). The recent increase in nutrients, especially nitrate, TP, and SRP in Lake Hayq, could be due to the expansion of agriculture (fertilizers, herbicides, and insecticides) along the lake's shore, siltation, and other organic pollution loads [47]. High phosphate levels are linked with the leaching of industrial waste water, agricultural wastes, and domestic wastes into the water system [48]. Since phosphorus and nitrogen in particular are thought to be the main factors limiting phytoplankton production in lakes [49]. Soil and rocks, municipal and industrial sewage, discharges from urban and agricultural drainage channels, runoff from animal manure storage areas, and the dumping of untreated sewage from villages and small settlements are sources of phosphorus in water bodies [50, 51]. Phosphate concentrations between 0.005 and 0.02 are the threshold limit for good water quality that sustains aquatic organisms [52].

Nitrogen is the limiting nutrient for algal growth in lakes and rivers. The principal source of nitrogen is agriculture [53]. Nitrogen can be entered into lakes and reservoirs via runoff, atmospheric deposition, or groundwater [54, 55]. Nitrogen fixation by plants and bacterial oxidation are also sources of nitrate [56]. The current nitrate concentration is found to be higher than the recommended value in drinking water for human health [45]. The lower water transparency and excessive growth of aquatic plants on the shore of the lake might have been caused by the recent nutrient enrichment.

### 3.3. Major Cations

In the present study, the overall mean and range of calcium, magnesium, sodium, and potassium were (24.17 mg/L; 16 to 35 mg/l), (54.33 mg/l; 54 to 55 mg/l), (36.17 mg/l; 24 to 52 mg/l), and (6.64 mg/l; 4.86 to 8.96 mg/l), respectively (Table 5). The concentration of the major ions varied with sampling sites. However, they met the WHO [45] drinking water guidelines. The recent major cations of the water quality result showed that Lake Hayq is a freshwater lake.

### 3.4. Major Anions

Bicarbonate is the dominant anion in surface water samples of Lake Hayq, with a concentration range of 212.4–342.87 mg/l. Its average concentration was 257.52 mg/l (Table 5). The sulfate content in the surface water samples varied between 69.4 and 173.1 mg/l, and the average value of sulfate of the present study was 131.8 mg/l (Table 5). These values were higher than the sulfate content in the atmospheric precipitation (2 ppm) [57].

In this study, chloride ion content in surface water samples ranged from 12.6 to 26.9 mg/l, with an average value of 18.68 mg/l (Table 5). The chloride value of Lake Hayq was within drinking water and aquatic life permissible limit [58].

In the present study, the overall mean and range of bicarbonate, sulfate, and chloride were (257.52 mg/l; 212.4–342.87 mg/l), (131.8 mg/l; 69.4 to 173.1 mg/l), and (18.68; 12.6–26.9 mg/l), respectively (Table 5). Similar to the major cations, the major ion analysis (bicarbonate and sulfate) also showed that the lake is freshwater [45, 58]. Bicarbonate is the dominant anion in the surface water samples from Lake Hayq.

### 3.5. Heavy Metals

The mean concentrations of the studied heavy metals in surface water were in the order of Pb > Fe > Cu > Zn > Ni > Cr > Mn (Table 6). In the present study, except for Pb, other heavy metals are within the permissible limit for drinking water quality standards [45]. Copper (Cu), chromium (Cr), zinc (Zn), nickel (Ni), and lead (Pb) are among the heavy metals that pose a hazardous effect on humans if their concentration exceeds their limit [59, 60].

Lake Hayq is a closed lake, without an outlet, and is surrounded by a mountainous landscape. Therefore, the probability of contamination by heavy metals might be very high. According to Pant et al. [14] and Singh et al. [13], lakes are susceptible to chemical contamination as they are stagnant compared to rivers and streams, which have more self-purification potential.

### 3.6. BOD_5_ and Total Coliforms

In the present study, the BOD_5_ varied among seasons, the highest (155.5 mg/l) and the lowest (2.67 mg/l) BOD_5_ were recorded in rainy and prerainy seasons, respectively. The highest (106 colony units/100 ml) and the lowest (42.99 colony unit/100 ml) total coliforms were recorded during rainy and prerainy seasons, respectively (Table 7). According to WHO [45], the BOD_5_ and the total coliform values were supposed to be <5 mg/L and 0 for drinking water purpose. This result suggests that Lake Hayq is polluted and not fit for drinking water purposes. Similar to the present study, microbial pollution due to total coliforms and fecal coliforms was reported for Lake Saheb Bandh in India [61]. The higher BOD_5_ and total coliforms during the rainy season might be associated with more organic pollutants from the catchment through runoff.

Most of the water quality parameters of Lake Hayq were within the WHO [45] maximum allowable concentration for drinking water. However, Pb (8.61 mg/l), BOD_5_ (77.75 mg/l), and total coliforms (72/100 ml water sample) were above the permissible limits, which were supposed to be <0.01 mg/l, <5 mg/l, and 0/100 ml for drinking water quality, respectively (Table 7). The suitability of water for drinking, aquatic life, and recreational uses depends on several factors. Therefore, efforts should be made to reduce their release into the lakes and rivers [62].

## 4. Conclusion and Recommendations

The water quality of Lake Hayq is being degraded from time to time due to the weak waste management system of lodges constructed on the shore of the lake, the absence of a buffer zone around the lake, and siltation through the River Ankerkeha that carries siltation to the lake.

Lake Hayq is less polluted based on the WHO drinking water quality guidelines. However, a few water quality parameters (lead, BOD_5,_ and total coliforms) are above the WHO drinking water quality permissible limit and need more attention. Thousands of people are using the lake water for drinking purposes and consuming fish from the lake. Lead might have been deposited in sediment and bioaccumulated in fish. Therefore, continuous water quality monitoring should be done, and the lake water should be treated before drinking. Further studies on microbial and heavy metal analysis in water and fish should be conducted along the food chain to see the level of biomagnification. In addition to this, impact on aquatic life and public health should be conducted.

## Figures and Tables

**Table 1:** Sampling site description.

Sampling sites	Characteristics	Depth (m)	Altitude (m)	Coordinate points (UTM)	
				*X*	*Y*
Littoral (SS1)	Near lodges (more pressure)	6.3	1903	575131.78	1252295.8
Agriculture (SS2)	Lake shore without buffer zone	5.6	1902	575561.74	1254135.79
Front (SS3)	Near degraded mountain	7.2	1904	579715.383	1254676.55
Ankerkeha (SS4)	With huge silt load	5.3	1900	579715.383	1253117.123
Control (SS5)	Relatively less impacted	10.1	1900	577842.94	1251695.23
Pelagic (SS6)	Open water	55.4	1907	576688.51	1252693.02

**Table 2:** Inorganic nutrient values (mean and SE) in six sites (SS1-littoral, SS2-agricultural, SS3-front, SS4-Ankerkeha, SS5-control, and SS6-pelagic).

Parameters	Sites						WHO standard (*µ*gL^−1^)
	SS1	SS2	SS3	SS4	SS5	SS6	
NH_3_ (*µ*gL^−1^)	25.72 ± 2.04	23.69 ± 2.04	32.68 ± 2.04	43.128 ± 1.76	25.09 ± 2.04	39.31 ± 2.04	—
NO_2_ (*µ*gL^−1^)	39.27 ± 4.41	4.81 ± 4.41	30.01 ± 4.41	56.13 ± 3.82	22.6 ± 4.41	48.16 ± 4.41	3000
NO_3_ (*µ*gL^−1^)	159.64 ± 7	167.91 ± 7	166.32 ± 7	164.98 ± 6.07	195.02 ± 7	165.72 ± 7	50000
SiO_2_ (*µ*gL^−1^)	194.41 ± 4.35	209.2 ± 4.35	202.96 ± 4.35	205.76 ± 3.77	208.35 ± 4.35	194.53 ± 4.35	—
TP (*µ*gL^−1^)	19.83 ± 1.72	7.43 ± 1.72	1.99 ± 1.72	21.76 ± 1.49	1.73 ± 1.72	11.58 ± 1.72	2000
SRP (*µ*gL^−1^)	1.14 ± 0.08	0.45 ± 0.08	0.64 ± 0.08	1.78 ± 0.07	0.46 ± 0.08	1.63 ± 0.08	2000

**Table 3:** Inorganic nutrient variation within seasons (prerainy, dry, rainy, and postrainy) in Lake Hayq.

Parameters	Seasons			
Nutrients	Prerainy	Dry	Rainy	Postrainy
NH_3_ (*µ*gL^−1^)	64.284 ± 2.036	23.619 ± 1.44	22.63 ± 1.44	35.406 ± 1.763
NO_3_ (*µ*gL^−1^)	155.883 ± 7.005	179.246 ± 4.953	168.497 ± 4.953	167.414 ± 6.066
SiO_2_ (*µ*gL^−1^)	183.842 ± 4.351	203.314 ± 3.076	199.335 ± 3.076	220.993 ± 3.768
TP (*µ*gL^−1^)	55.082 ± 1.719	5.131 ± 1.216	2.034 ± 1.216	1.616 ± 1.489
SRP (*µ*gL^−1^)	3.535 ± 0.084	0.871 ± 0.059	0.499 ± 0.059	0.307 ± 0.073

**Table 4:** Biophysiochemical water quality parameters of Lake Hayq compared with other lakes and WHO maximum allowable concentration for the drinking water guideline.

Parameters	Present study	[43]	[33]	[41]	[45]
pH	8.6	6.5–8.05	9	6.98–9.97	6.5–9.5
Temperature (°C)	23.5	24.9–30.8	ND	16.4–31.3	15–29.4
DO (mg/L)	7.5	6.5–7.4	3–8.4	ND	>5
Turbidity (NTU)	4	ND	ND	51–989	<5
Conductivity (*µ*S/cm)	887.8	ND	ND	60–1000	<1000
TDS (mg/L)	676.74	116–183.3	ND	20–500	1000
Total alkalinity (meqL^−1^)	9.3	2.1–3.27	9.8	0.42–72.6	ND
Nitrate (*µ*gL^−1^)	90.9	5200–8600	40	3–4700	45000
Nitrite (*µ*gL^−1^)	81.1	50–230	10	0–3250	500
NH4^+^ (*µ*gL^−1^)	42.7	700–2230	260	ND	2000
Na^+^ (mg/L)	36.17	ND	61.2	ND	358
K^+^ (mg/L)	6.64	ND	4.2	ND	12
Ca2^+^ (mg/L)	24.17	ND	1.02	ND	200
Mg2^+^ (mg/L)	54.33	ND	97.8	ND	150
PO_4_^3−^ (*µ*gL^−1^)	8.3	0.13–0.46	22	ND	2000
^∗^Pb (lead)	8.61	ND	ND	ND	0.01
F^−^ (mg/L)	0	0.3–1.3	1	ND	1.5
Cl^−^ (mg/L)	18.68	35–70	35.8	ND	5
CO_3_^−^ 2 (mg/L)	8.82	ND	24	ND	ND
HCO_3_^−^ (mg/L)	257.52	ND	292.8	ND	ND
SO4^2−^ (mg/L)	131.8	ND	2.8	ND	400
^∗^BOD_5_	77.75	ND	ND	ND	5
^∗^Total coliforms	71.25	ND	ND	ND	0

ND: no data. Parameters with ^∗^indicate that they do not meet the standard.

**Table 5:** Results of chemical analyses of major cations, anions TH (total hardness), and TDS (mg/L).

Site	Ca^2+^	Mg^2+^	K^+^	Na^+^	NH_4_^+^	Cl^−^	SO_4_^2−^	HCO_3_^−^	CO_3_^−^	F^−^	TH	TDS
SS1	16	54	6.54	24	0.001	12.6	144.6	212.4	5.92	0.003	265	676.74
SS2	23	55	4.86	36	0.002	18.6	69.4	342.87	6.86	0.003	286.7	676.74
SS3	26	55	5.22	33	0.001	15.4	163.5	248.99	7.75	0.002	294.2	675.32
SS4	35	54	7.15	41	0.001	22.5	168.1	236.78	18.26	0.002	312.4	675.32
SS5	22	54	8.96	52	0.002	26.9	173.1	236.8	7.09	0.001	280	677.45
SS6	23	54	7.11	31	0.001	16.1	71.87	267.3	7.01	0.003	282.4	678.88
Min	16	54	4.86	24	0.001	12.6	69.4	212.4	5.92	0.001	265	675.32
Max	35	55	8.96	52	0.002	26.9	173.1	342.87	18.26	0.003	312.4	678.88
Mean	24.17	54.33	6.64	36.17	0.00	18.68	131.8	257.52	8.82	0.00	286.78	676.74
Sta.Dev	6.24	0.52	1.49	9.58	0.00	5.23	48.33	45.49	4.66	0.00	15.82	1.35

**Table 6:** Results of heavy metal analyses of the surface water samples of the study area (concentrations in mg/l).

Site		Fe	Mn	Cr	Cu	Pb	Zn	Ni
Surface water	SS1	0.54	0.01	0.02	0.56	4.11	0.00	0.00
	SS2	0.59	0	0	0.52	20.1	0.581	0.02
	SS3	0.61	0	0	0.41	2.1	0.00	0.00
	SS4	0.72	0	0	0.41	6.8	0.119	0.09
	SS5	0.85	0	0	0.92	11.3	0.00	0.00
	SS6	0.43	0	0	0.26	7.26	0.41	0.00

Min		0.43	0	0	0.26	2.1	0	0

Max		0.85	0.01	0.02	0.92	20.1	0.581	0.09

Mean		0.62	0.002	0.003	0.51	8.61	0.185	0.018

Sta. dev		0.15	0.004	0.008	0.23	6.438	0.251	0.036

**Table 7:** Total coliforms and BOD_5_ variation among the four seasons in Lake Hayq.

Parameters	Prerainy	Dry	Rainy	Postrainy
Total coliforms	42.985	47.835	77.75	47.43
BOD_5_	2.6675	3.4425	41.14	27.5

## Data Availability

Data will be available on request.
